# Surface Crystallization of a MgO/Y_2_O_3_/SiO_2_/Al_2_O_3_/ZrO_2_ Glass: Growth of an Oriented β-Y_2_Si_2_O_7_ Layer and Epitaxial ZrO_2_

**DOI:** 10.1038/srep44144

**Published:** 2017-03-10

**Authors:** Wolfgang Wisniewski, Sabrina Seidel, Christian Patzig, Christian Rüssel

**Affiliations:** 1 Otto-Schott-Institut, Jena University, Fraunhoferstr. 6, Jena 07743, Germany; 2 Fraunhofer Institute for Microstructure of Materials and Systems IMWS, Walter-Huelse-Straße 1, Halle (Saale) 06120, Germany

## Abstract

The crystallization behavior of a glass with the composition 54.7 SiO_2_·10.9 Al_2_O_3_·15.0 MgO·3.4 ZrO_2_·16.0 Y_2_O_3_ is studied using X-ray diffraction (XRD), scanning electron microscopy (SEM) including electron backscatter diffraction (EBSD) and (scanning) transmission electron microscopy [(S)TEM] including energy-dispersive X-ray spectrometry (EDXS). This glass shows the sole surface crystallization of four different yttrium silicates of the composition Y_2_Si_2_O_7_ (YS). The almost simultaneous but independent nucleation of α-, β-, δ-, and ε-YS at the surface is followed by growth into the bulk, where ε-YS quickly dominates a first crystallized layer. An accumulation of Mg at the growth front probably triggers a secondary nucleation of β-YS, which forms a thin compact layer before fragmenting into a highly oriented layer of fine grained crystals occupying the remaining bulk. The residual glass between the YS growth structures allows the crystallization of indialite, yttrium stabilized ZrO_2_ (Y-ZrO_2_) and very probably μ-cordierite during cooling. Hence, this glass basically shows the inverted order of crystallization observed in other magnesium yttrium alumosilicate glasses containing less Y_2_O_3_. An epitaxial relationship between Y-ZrO_2_ and ε-YS is proven and multiple twinning relationships occur in the YS phases.

In the past few years, glasses and glass-ceramics of the system MgO/Al_2_O_3_/SiO_2_ have gained a great deal of interest, mainly due to their good mechanical properties such as high hardness, high Young’s moduli, high strength and a high fracture toughness^1^,^2^,^3^,^4^,^5^ as well as to their interesting dielectric properties^6^,^7^,^8^. These glass-ceramics have frequently been proposed for various applications such as hard disc substrates^9^, millimeter-wave dielectrics^6^,^7^ and dental materials^3^. Most glasses in this system predominantly crystallize from the surface, as for example in the case of glass with the stoichiometry of cordierite (2 MgO · 2 Al_2_O_3_ · 5 SiO_2_)^10^,^11^,^12^,^13^. In this case, the surface crystallization of μ-cordierite (also denoted as the high-quartz solid solution) is observed at temperatures of ca. 900 °C, which irreversibly transforms to indialite (also denoted as α-cordierite) after longer crystallization times of ca. 12 h at temperatures above ca. 950 °C. This transformation is accelerated at higher temperatures^13^. Bulk crystallization may be induced by additives such as TiO_2_^14^,^15^,^16^,^17^, ZrO_2_^1^,^2^,^3^,^4^,^5^,^18^,^19^ or both^20^,^21^ which act as nucleating agents.

The effects of adding 0.5 to 5.0 mol% Y_2_O_3_ to this system have recently been analysed^4^,^5^,^22^. Concentrations of 2.4 mol% prevent the precipitation of the high-/low- quartz solid solution (high-/low-QSS)^4^,^5^,^22^, which were observed in Y_2_O_3_-free glass-ceramics^1^,^2^,^3^,^4^,^5^,^18^,^19^. As described in the refs 4 and 5, holding a glass with the mol% composition 50.6 SiO_2_·20.7 MgO·20.7 Al_2_O_3_·5.6 ZrO_2_·2.4 Y_2_O_3_ at the nucleation temperature of 950 °C for 5 h, subsequently cooling it to room temperature (RT) and then crystallizing it at 1060 °C for 1 h did not result in the formation of any QSS, but spinel (MgAl_2_O_4_), ZrO_2_ and considerable quantities of residual glass were detected. Due to the high coefficient of thermal expansion (CTE) of the low-QSS (CTE_20–300_ _°C_ = 13.2 · 10^−6^ K^−1^)^23^ as well as the strong volume contraction accompanying the phase transition from the high- to the low-QSS during cooling^2^,^21^, high mechanical stresses occur in these glass-ceramics^2^,^3^,^21^. This apparently results in advantageous mechanical properties. Surprisingly, the glass-ceramics containing 2.4 mol% Y_2_O_3_ also show high mechanical strengths although the low-QSS is not detected, which might be explained by the formation of spinel (CTE_20–800_ _°C_ = 8 · 10^−6^ K^−1^)^23^ and ZrO_2_ in its tetragonal or cubic form (CTE_tetragonal_ = 10.5 · 10^−6^ K^−1^)^24^ which also show large CTE values^4^. Furthermore, indialite was detected at the surface of these glass-ceramics and the crystallization of several different Y_2_Si_2_O_7_ (YS) phases was obtained after longer crystallization at the second annealing step and various crystallization regimes^25^. In a previous experiment, the chemical composition of the residual glassy phase in the glass-ceramic was analyzed by scanning transmission electron microscopy (STEM) including energy-dispersive X-ray spectroscopy (EDXS) after crystallization at 950 °C for 5 h and 1060 °C for 1 h. Subsequently, a glass with this composition was melted and investigated^25^. In contrast to the parent glass, this glass shows the surface crystallization of different YS-phases and indialite, while bulk nucleation does not occur^25^.

By analogy, many compositions in the Y_2_O_3_/Al_2_O_3_/SiO_2_ system show the crystallization of various crystallographically different YS phases and yttrium aluminum garnet (YAG)^26^,^27^,^28^,^29^. At least seven YS phases have been described in the literature so far^30^,^31^ and a small overview of the literature was recently presented in ref. 29. An additional phase associated with the unindexed JCPDS-file 21-1459 was denoted as “z-Y_2_Si_2_O_7_” and described as a “lowest temperature phase” in ref. 32. Table 1 shows the notations of various phases with the composition Y_2_Si_2_O_7_ and some correlating database entries. A “y-phase” is also inconsistently mentioned as illustrated in ref. 29 and hence not noted in Table 1. Please see ref. 29 for a more detailed discussion of this Table.

It has been stated that α-YS is the stable phase below 1225 ± 10 °C and above transforms to β-YS which is stable up to 1445 ± 10 °C where it transforms to γ-YS and further to δ-YS at 1535 ± 10 °C^31^. However, β-, δ- and ε-YS have been shown to simultaneously occur in some YAG-containing glass-ceramics^26^,^27^,^28^,^29^ while α-YS was additionally observed in glass-ceramics grown from an SiO_2_-MgO-Al_2_O_3_-ZrO_2_-Y_2_O_3_ glass with a higher Al content^25^. These results also illustrated that an X-ray diffraction (XRD)-analysis of these phases is problematic if they occur simultaneously due to multiple peak super positions, a problem illustrated in detail in ref. 29. For instance, XRD-patterns obtained from some glass-ceramics have been interpreted to indicate α-YS, not found using electron backscatter diffraction (EBSD), instead of the three other yttrium silicates proven to exist in the samples^27^. γ-YS has been classified as quasi ductile ceramic thermochemically compatible with SiC and SiO_2_ and with a potential to improve mechanical properties^33^. The ζ-YS^30^ was obtained as a by-product during the production of yttrium oxotellurates, while the η-YS described in ref. 31 is a triclinic high pressure phase obtained as a by-product of experiments in the system Na_2_O-Y_2_O_3_-SiO_2_. Twinning has been indicated in the triclinic α-YS^29^ and the moniclinic β-YS^28^. A five-fold symmetry due to the microtwinning β-YS in the presence of growth barriers has been reported^28^.

This article provides a study on the crystallization of a glass with the mol% composition 54.7 SiO_2_·10.9 Al_2_O_3_·15.0 MgO·3.4 ZrO_2_·16.0 Y_2_O_3_, which is the composition of a residual glass phase in glass-ceramics produced from a glass of the mol% composition 50.6 SiO_2_·20.7 MgO·20.7 Al_2_O_3_·5.6 ZrO_2_·2.4 Y_2_O_3_^4^,^25^. This glass does not nucleate in the bulk^25^, but shows sole surface crystallization of four different YS-phases. Secondary phases including indialite crystallize in the residual glass between the YS crystals. The crystallization behavior, preferred crystal orientations and epitaxial relationships are analyzed in detail by scanning electron microscopy (SEM) including EBSD and analytical STEM including EDXS. The results are discussed in the context of growth mechanisms in glasses, cordierite surface crystallization and Y_2_Si_2_O_7_ crystallization. A complete growth model for the prepared glass-ceramics is presented.

## Results

**Figure 1:**
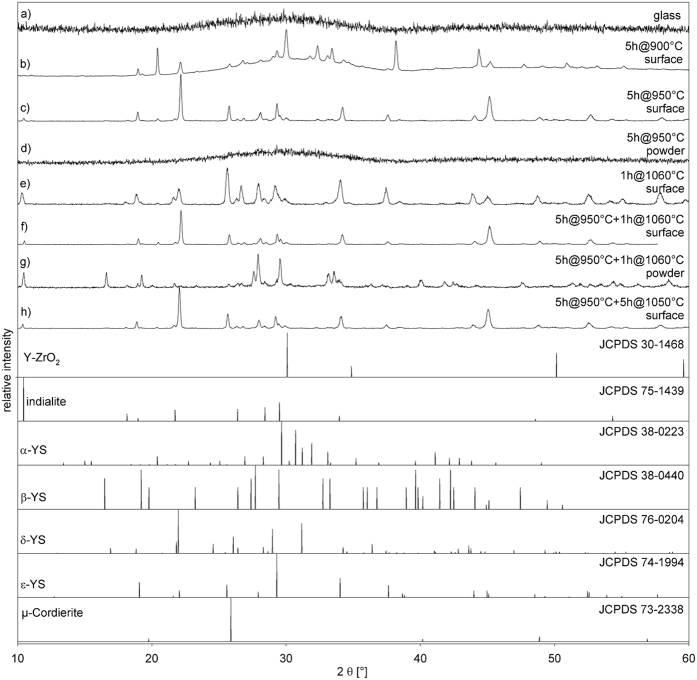
XRD-patterns acquired from (**a**) the produced glass and (**b–h**) from the surfaces of compact samples or powders of glass-ceramics annealed according to the stated annealing regimes. The theoretical patterns of phases possibly occurring in these glass-ceramics are presented below for comparison.

Although the problems of analyzing samples containing multiple phases of the composition Y_2_Si_2_O_7_ have been illustrated^25^,^26^,^27^,^28^,^29^, XRD-patterns acquired from various glass-ceramic samples annealed in this system are presented in Fig. 1 along with the theoretical patterns of phases indicated in this system by EBSD-analyses which will be presented later. As expected, the produced glass is X-ray amorphous as indicated by pattern (a). Pattern (b) was recorded from the surface of a sample crystallized at 900 °C for 5 h. All peaks in this pattern are attributable to at least one of the YS phases. It is noteworthy that there is no peak at 2θ = 10.4° in contrast to all the patterns acquired from surfaces annealed at higher temperatures. While pattern (c), also recorded directly from the surface of a compact sample, shows discrete peaks after annealing at 950 °C for 5 h, the pattern (d) obtained after powdering part of the same sample does not. This indicates a pure surface crystallization in this system and that the crystallized surface layer did not contain enough crystalline volume to be detected after mixing it with the uncrystallized glass of the bulk during powdering. Although most of the peaks may be attributed to multiple phases, some may be clearly identified in these XRD-patterns if the phase selection is limited to those featured in Fig. 1. Indialite is proven to exist in the patterns (c) and (e–h) by the characteristic peak at 2θ = 10.4° and a weak peak at 18.1°. Pattern g) contains clearly attributable peaks at 2θ = 16.5° and 47.4° (β-YS), as well as at 2θ = 36.4° (δ-YS). A peak at 2θ = 57.7° is solely attributable to ε-YS. While the peaks of yttrium stabilized zirconia (Y-ZrO_2_) are not superimposed by any other phase, none of the XRD-patterns clearly indicate the presence of this phase. The peak intensities always differ between the patterns acquired from the surface of a compact sample and the powder of the same glass-ceramic, but as this may be caused by textures of specific phases as well as by layers of crystals, reliable conclusions cannot be based on these XRD-patterns alone. Although spinel was located in the parent glass-ceramic^25^, it could not be proven to occur in the glass-ceramics analyzed here. All XRD-peaks attributed to spinel are superimposed by at least one other phase proven to occur in these samples by the EBSD and TEM analyses presented below.

### Surfaces of Crystallized Samples

**Figure 2:**
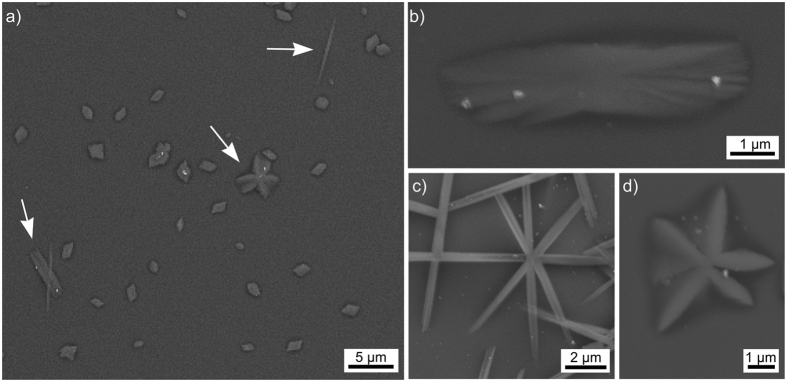
(**a**) SEM-micrograph of the immediate surface after crystallization for 5 h at 900 °C. Arrows highlight specific growth structures. More detailed growth structures of (**b**) β-YS, (**c**) δ-YS and (**d**) ε-YS are presented below.

Annealing this glass for 5 h at 860 °C, i.e. only 20 K above the glass transition temperature T_g_ of 840 °C^25^, did not lead to any discernible signs of crystallization. After 5 h at 900 °C, the surface was partially covered by crystals with different morphologies as shown in Fig. 2(a). The large majority of crystals are more or less rhomboids, ca. 1 μm in diameter, and EBSD-patterns acquired from them may be reliably indexed as triclinic α-YS, see also Table 1. The 180° misorientation already noted in ref. 29 frequently occurs, pointing towards a twinning relationship within this phase. The arrows in Fig. 2(a) highlight crystals with differing but characteristic morphologies of rectangles, needles and stars. Examples of these are presented in greater detail and, based on EBSD-measurements, they may be attributed to (b) monoclinic β-YS, (c) orthorhombic δ-YS and (d) monoclinic ε-YS. While the rectangles of β-YS at this surface only provide low quality EBSD-patterns making indexing a bit challenging, the δ- and ε-YS crystals provide high quality patterns which enable a more detailed analysis. The central δ-YS structure shows eight needles and EBSD-measurements show that the [100]-direction, i.e. the long a-axes, are always oriented parallel to the long axes of the needles. This primary growth direction is in agreement with the growth of δ-YS in a different glass where the crystals were forced to continuously change their growth direction due to barriers in the matrix^28^. The [100]-direction is also parallel to the surface, increasing the probability that these crystals are truly needles and not slabs and also indicating an oriented nucleation of this phase with the a-axes parallel to the surface. By contrast, the entire ε-YS structure in Fig. 2(d) only shows one orientation pointing towards a dendritic growth mechanism of this phase under the given conditions. Other phases could not be located in this glass-ceramic and the tiny bright particles in Fig. 2 did not provide EBSD-patterns, i.e. they are most probably dust on the surface.

**Figure 3:**
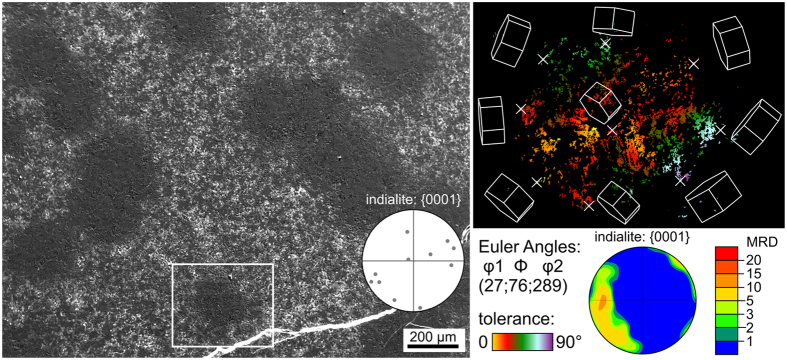
SEM-micrograph of the immediate surface after the sample was crystallized at 950** **°C for 5** **h, cooled to RT and then annealed another 1 h at 1060 °C. The {0001}-PF of indialite contains the pole positions of 11 single EBSD-patterns acquired from different circular areas in the micrograph. An orientation map containing only data points attributed to indialite in an EBSD-scan performed on the framed area is presented to the right along with the wire frames of indialite unit cells of selected positions marked by crosses. The {0001}-PF of a texture calculated from this data set is also presented.

Figure 3 presents an overview of a glass-ceramic surface representative for all annealing regimes including temperatures of 950 °C or higher. It contains circular regions of relatively smooth, compact surface crystallization separated by rough regions with a strong topography and hence edge effects which appear bright. Some large cracks are also observed (bottom). An EBSD-scan was performed in the framed area with a step size of 800 nm. While EBSD-patterns attributable to each of the phases noted in Fig. 1 (except μ-cordierite) could be obtained from this surface, this step size is not suited to resolve the details of the crystal structures as some of them are less than 1 μm in diameter. However, an orientation map of this scan containing only data points reliably attributed to indialite is presented in Fig. 3 to show that all the indialite domains within this circular structure are related to a single crystal orientation when allowing a tolerance of 90°. The dominant orientation preference of a c-axis parallel to the sample surface is illustrated by the presented {0001}-PF of a texture calculated from the dataset and quantified in multiples of a random distribution (MRD). Although the large tolerance of 90° in the orientation map incorporates a broad variety of orientations, the wire frames of the unit cells of specific orientations also presented in Fig. 3 illustrate that the orientations change systematically and continuously from the center outwards. Generally the orientation changes so that the short c-axis of indialite becomes increasingly parallel to the radius of the circular structure and hence parallel to the main growth direction and the surface. The most probable reason for this observed orientation relationship is that the indialite within the entire scanned area originated from only one nucleus. However, links between the individual indialite domains are neither observed in the EBSD-data set nor in the SEM-micrograph.

This observation of one dominant orientation and its continuous change was observed in every analyzed circular domain. Hence one EBSD-pattern obtained near the center may be assumed to represent an orientation relatively close to the nucleus of each respective domain. The {0001}-PF in Fig. 3 contains the poles of 11 indialite EBSD-patterns acquired from different circular domains in the SEM-micrograph of Fig. 3. While eight indicate orientations with the c-axis almost parallel to the surface, three do not. Hence, it seems that indialite preferably crystallizes with the c-axis parallel to the sample surface.

**Figure 4:**
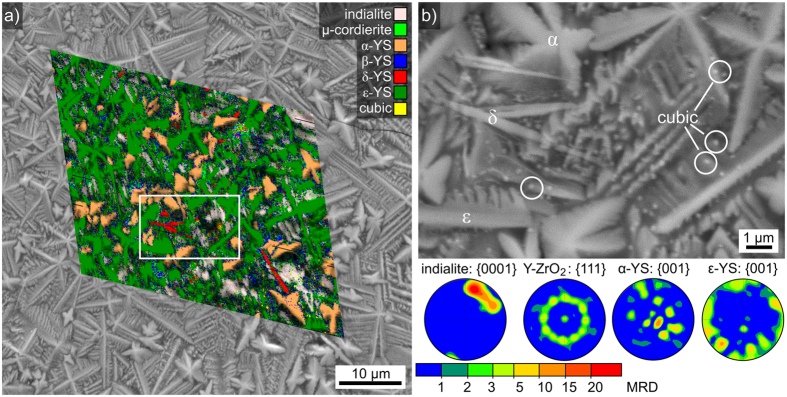
(**a**) SEM-micrograph of the immediate surface after the same annealing procedure featured in Fig. 3. It is superimposed by the phase map of a performed EBSD-scan. The framed area is presented in detail in the SEM-micrograph (**b**) which illustrates the morphology of the clearly discernible phases α-YS, δ-YS, ε-YS and the cubic phase. Pole figures of textures calculated from the data of selected phases are presented below.

The detailed morphology of the crystals at this surface is illustrated by the SEM-micrographs of a two-step crystallized glass-ceramic in Fig. 4. It is again representative for all surfaces resulting from the annealing regimes including temperatures of at least 950 °C performed for this article. The overview in Fig. 4(a) is superimposed by the phase + image quality (IQ)-map of a detailed EBSD-scan performed on the area with a step size of 150 nm. While most of the surface structures are attributed to indialite and α- or ε-YS, reliably indexable patterns^25^ could also be obtained for β- and δ-YS, as well as a cubic phase. A few data points in the scan were also attributed to μ-cordierite (a.k.a. the “high-quartz solid solution”^25^ or the “β-quartz solid solution”^12^,^34^) but single patterns clearly proving the presence of this phase have not been obtained from any glass-ceramics produced from this glass so far.

μ-cordierite should not occur after annealing at 1060 °C because it irreversibly transforms to indialite after crystallization at 950 °C for 12 h^13^. This phase transition is completed within a few minutes at temperatures above 1010 °C in stoichiometric cordierite glass-ceramics^13^. Hence μ-cordierite may occur in glass-ceramics crystallized at 900–950 °C, but any glass-ceramics crystallized at 1060 °C should only contain indialite due to a complete phase transformation. However, it is not impossible that some μ-cordierite crystallizes during cooling.

The framed area is presented in detail in Fig. 4(b) to visualize the distinct growth morphologies of the phases at these surfaces. Due to the higher annealing temperature compared to the structures in Fig. 2, α-YS now grew in the form of dendrites with barely discernible secondary structures instead of the rhomboids observed after growth at 900 °C. The δ-YS shows the same needles already observed in Fig. 2(c) and ε-YS shows rather classic dendritic growth with more clearly pronounced secondary structures than those discernible in Fig. 2(d). The cubic phase occurs in the form of the very small, bright particles circled in white. As they provide clear EBSD-patterns they are not of the same origin as the particles observed in Fig. 2. However, their chemical composition cannot be reliably determined by energy dispersive X-ray spectroscopy (EDS) in an SEM due to their small size.

**Figure 5:**
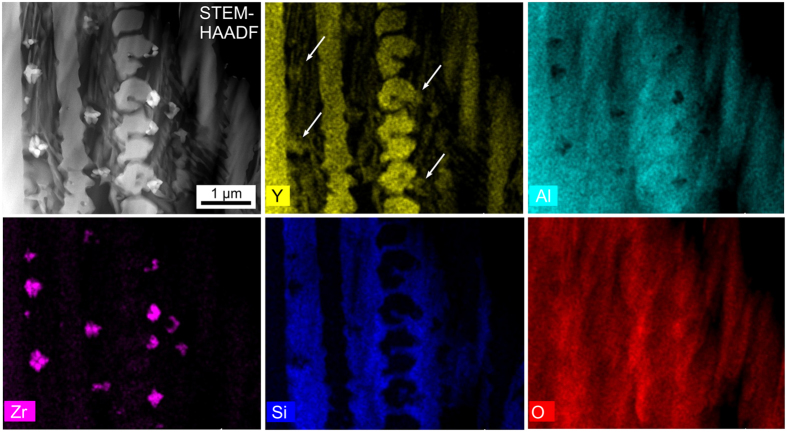
STEM-HAADF micrograph of the dendritic microstructure and the corresponding element maps of Y, Al, Zr, Si, and O.

Hence a sample of this glass-ceramic was prepared for analysis in a STEM. A HAADF-micrograph of the microstructure in a cross section just below the surface and corresponding element maps gained with EDS parallel to the micrograph acquisition are presented in Fig. 5. Zr is clearly concentrated in the bright particles (smaller than 300 nm in diameter) adjacent to the dendritic structures which are enriched in Y and should hence be composed of either the α- or the ε-YS observed in dendritic morphology in Fig. 4(b). However, the Zr-enriched particles also contain Y (arrows), indicating the cubic phase detected by EBSD to be yttrium stabilized zirconia (Y-ZrO_2_). Quantifying the Y-ZrO_2_ composition under the assumption that it only contains Y, Zr and O leads to a Zr:Y ratio of 73:27 which is in acceptable agreement with Y-ZrO_2_ described in the literature^35^. Al is concentrated outside of the YS and Y-ZrO_2_ while Si and O show a relatively homogenous distribution. As should be expected, Si does not occur in the Y-ZrO_2_ particles.

Pole figures (PFs) of textures calculated for selected phases in the EBSD-scan featured in Fig. 4(a) are also presented in Fig. 4. The {0001}-PF of indialite is similar to the one presented in Fig. 3. The orientation distribution is less broad (deviations of only 38°) because this scan does not cover an entire circular domain. The PF of the texture calculated for Y-ZrO_2_ indicates a clear texture with a {111}-plane parallel to the surface and more or less random rotations around its normal. This PF is based on 243 data points attributed to 45 grains containing at least three adjacent data points. It seems probably that the ring in the PF would become more homogeneous with an increasing number of grains, but data acquisition is not trivial as only ≈0.003% of the data points are attributed to this phase when using the applied step size of 150 nm. The PF of α-YS does not indicate a texture, but the 180° misorientation observed in some crystals after annealing at 900 °C occurs in almost every α-YS grain of Fig. 4a. As twinning is often a mechanism of stress relaxation, it seems reasonable that twinning would occur more often in this environment almost completely crystallized with multiple phases than when the crystals are well separated by a glass matrix (see Fig. 2a). The PF of ε-YS shows that crystal orientations with the c-axis parallel to the surface preferably occur within the scanned area. The data sets of β- and δ-YS in the EBSD-scan contain too few grains for any acceptable texture analysis and the existence of μ-cordierite at this surface is considered unlikely as discussed above.

### Cut planes through crystallized samples

**Figure 6:**
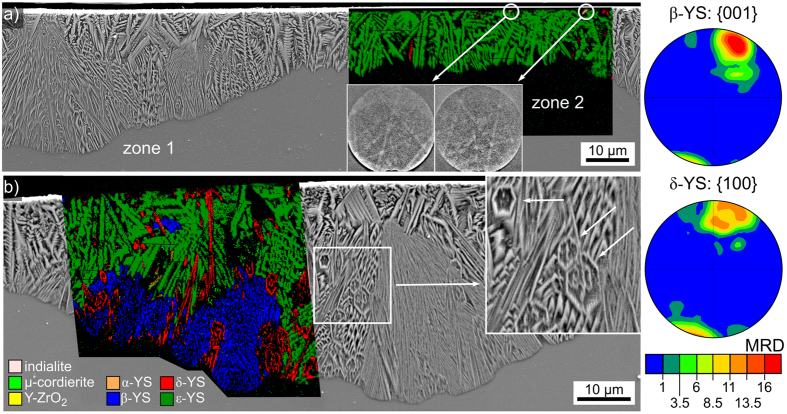
SEM-micrographs of the surface crystallized layer in a cross section after crystallization at 950 °C for 5** **h superimposed by phase maps of EBSD-scans performed on the respective areas. (**a**) Zone 1 and zone 2 with a phase-map focused on zone 2 which only contains ε-YS with a few δ-YS grains. The two single patterns obtained near the edge to the initial surface originate from α-YS. (**b**) Focus on zone 1 which contains significant amounts of β-YS and δ-YS in addition to ε-YS. PFs of textures calculated for the datasets of β- and δ-YS in this scan are presented to the right.

Cross sections of samples annealed by various regimes were prepared to analyze the crystal growth into the bulk. In agreement with ref. 25 and Fig. 1, surface crystallization was solely observed in all samples, i.e. bulk nucleation was never detected. The edge to the initial sample surface is located at the top of all cross sections so that the primary direction of crystal growth is always from top to bottom in the respective figures. Figure 6 illustrates the crystallized layer in a sample annealed at 950 °C for 5 h.

Figure 6(a) shows that the crystal layer contains two different growth zones: zone 1 is thicker and of more diverse growth morphology while zone 2 is thinner and mainly shows dendritic crystal morphologies. The superimposed phase + IQ-map shows that zone 2 is predominantly composed of ε-YS, although some δ-YS is also detected. The two presented EBSD-patterns were acquired in the circled areas at the edge to the initial surface and can be reliably indexed as α-YS, which is in agreement with the results presented in Fig. 4. The crystallization in zone 1 is presented in greater detail in Fig. 6(b): dendritic growth occurs next to very fine grained areas which extend furthest into the bulk. The framed area is presented in greater detail in the inset to show that another growth morphology including six-sided rings is also discernible. The phase + IQ-map of an EBSD-scan superimposed on the SEM-micrograph shows that the dendritic structures (dark green) are again attributed to ε-YS. The fine grained, fast growing structures (blue) are composed of β-YS while the six-sided rings (red) are attributed to δ-YS. Similarly channeled growth of δ-YS has previously been observed in the bulk of a YAG-containing glass ceramic^28^.

Orientations of the dendritic ε-YS with the c-axis somewhat parallel to the surface seem to be slightly preferred in the presented data set in agreement with Fig. 4, but the texture is not strong enough to clearly indicate a growth selection within the scanned area. PFs of textures calculated for β- and δ-YS are presented in Fig. 6. Both phases show clearly preferred orientations with respect to the main growth direction, i.e. perpendicular to the initial sample surface: for β-YS the c-axes and for δ-YS the a-axes are preferably aligned parallel to the main growth direction.

**Figure 7:**
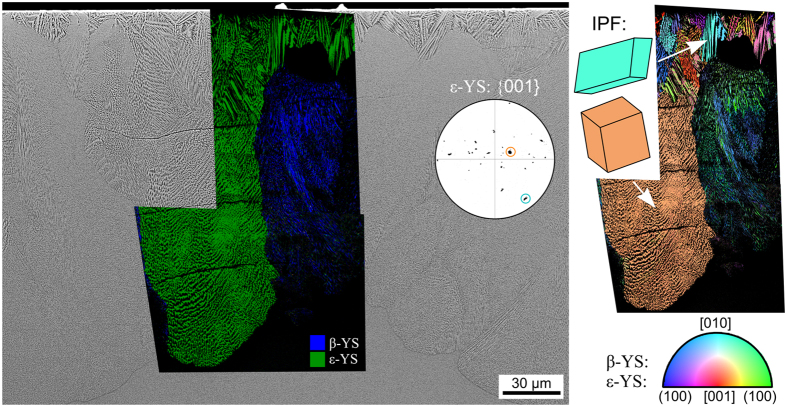
SEM-micrograph obtained from the surface crystallized layer in a cross section after crystallization at 950** **°C for 10** **h superimposed by the phase + IQ-maps of EBSD-scans performed on the area. The IPF + IQ-maps of these scans are presented to the right along with unit cells indicating selected ε-YS orientations. These orientations are also highlighted by colored circles in the {001}-PF of ε-YS which is presented to further visualize the orientations of this phase occurring within the scanned area.

Increasing the annealing time to 10 h at the same temperature of 950 °C led to the 150–220 μm thick crystallization layer shown in Fig. 7. The uneven growth front indicates either multiple growth velocities or that growth did not begin at the same time. The superimposed phase + IQ-maps of performed EBSD-scans show that the dendritic ε-YS is generally limited to a surface layer of less than 50 μm, although a large structure of ε-YS prevails up to the growth front in this area. The bulk of the crystallized layer is composed of a layer of very fine crystal structures which do not provide high quality EBSD-patterns but are generally index as β-YS. Very small amounts of δ-YS were detected close to the growth front.

The IPF + IQ-maps of the same EBSD-scans are presented to the right and show that the surface layer of ε-YS is composed of multiple dendrites with multiple orientations while the structure growing into the bulk shows one dominant orientation. The {001}-PF of ε-YS is presented to show that orientations with the short c-axis more or less parallel to the surface predominantly occur in agreement with previous observations, see Figs 4 and 6. Wire frames of two selected orientations are presented and their respective orientations are marked in the PF by colored circles. The dominant ε-YS structure grows with its c-axis almost parallel to the surface while the turquoise colored dendrite shows an orientation with the c-axis almost perpendicular to it. The sometimes multiple orientations indicated within the ε-YS dendrites are the result of an indexing problem: two pseudosymmetric orientations receive the same number of votes, leading to a confidence index (CI) of exactly 0.000. Almost 47% of the data points attributed to ε-YS in the presented data receive a CI value of exactly 0.000, excluding the CI as a reliable filter for this phase. By comparison, 72% of the data points attributed to ε-YS receive a fit factor <1° which is an alternative indicator for a correctly indexed EBSD-pattern.

**Figure 8:**
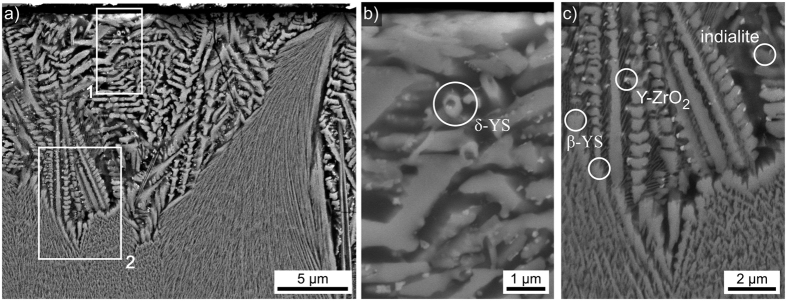
SEM-micrographs obtained from the surface crystallized layer in a cross section after crystallization at 950** **°C for 5** **h, cooling to RT and then annealing another 1 h at 1060 °C. EBSD-patterns indexable as the respectively stated phases were obtained from the circled growth structures. (**a**) Overview of layer 1 and the boundary to layer 2, the framed areas 1 and 2 are presented in greater detail to the right. (**b**) Detailed SEM-micrograph of the cross section near the edge to the initial surface (top). (**c**) Detailed SEM-micrograph of the boundary between layer 1 and layer 2.

Annealing this glass at 950 °C for 5 h, cooling it to RT and annealing it again at 1060 °C for 1 h led to the cross section microstructure presented in Fig. 8(a). This annealing regime was chosen to enable a comparison with a glass analyzed in ref. 25 where the same two-step process was reported to be decisive for the mechanical properties. In analogy to Fig. 7, two layers are observed: dendritic growth adjacent to the surface and fine grained crystals in layer 2. However, the microstructure is somewhat coarser than in the samples only annealed at 950 °C which enables a better analysis of the microstructure details. Figure 8(a) illustrates that layer 2 sometimes originates at the original surface itself which is in agreement with the β-YS detected at the immediate surface in Fig. 2. The area in frame 1 is presented in greater detail in Fig. 8(b) to visualize the growth morphology of a small crystallite near the surface. An EBSD-pattern acquired from the circled area was reliably indexed as δ-YS. The area in frame 2 is presented in greater detail in Fig. 8(c) to illustrate the boundary between the crystallization layers 1 and 2 in greater detail. While the main dendritic structure was shown to be composed of ε-YS in Fig. 6, single EBSD-patterns acquired from the circled areas show that the indialite and Y-ZrO_2_ observed at the immediate surface in Fig. 4 also occur in the bulk. Layer 2 is composed of β-YS which forms an almost compact layer at the boundary to layer 1 but assumes the fine grained microstructure already described in Figs 6(b) and 7 during further growth into the bulk.

**Figure 9:**
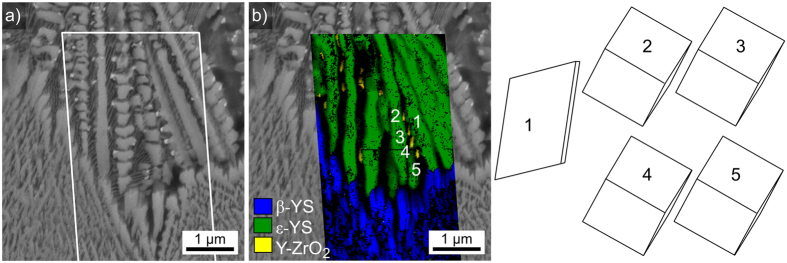
Detailed analysis of the boundary between the growth layers 1 and 2 presented in Fig. 8c. (**a**) SEM micrograph, please note the small, bright phase at the tips of the dendrites. The framed area was scanned by EBSD. (**b**) phase + IQ-map of the performed EBSD-scan. The presented unit cells visualize the orientations of 1: the dendritc arm of ε-YS and 2–5: the respective Y-ZrO_2_ particles.

The SEM-micrograph in Fig. 9(a) shows the same area as Fig. 8(c) with a frame illustrating where an EBSD-scan was performed. It also shows that the interdendritic area is not homogeneous but contains two phases, i.e. either two phase separated residual glasses or one residual glass (dark) and a crystal phase too small to be analyzed in the SEM using EDS or EBSD. However, a quantitative STEM-EDXS analysis of the interdendritic space free of Y in Fig. 5 resulted in a composition of Mg_6.8_-Al_15.4_-Si_17.5_-O_60.3_ (at%) in these areas, which is very close to the composition of cordierite (Mg_2_Al_4_Si_5_O_18_) which corresponds to Mg_6.9_-Al_13.8_-Si_17.2_-O_62.1_ at%. Since indialite crystals were confirmed in this layer in Fig. 8(c), the bright phase in the interdendritic space is most probably also indialite, but with crystals too small to enable EBSD-pattern acquisition.

The phase + IQ-map of the scan is superimposed on the micrograph in Fig. 9(b): as expected, the dendritic growth structures are attributed to ε-YS and the crystals in layer 2 to β-YS. The very small, bright particles at some of the dendritic tips are attributed to Y-ZrO_2_. The wire frames of unit cells representing the crystal orientations of 1: ε-YS and 2–5: the Y-ZrO_2_ are presented below. As discernible in Fig. 9(a), all these cubic particles formed at the tips of the same dendrite and the wire frames illustrated that they all show the same orientation, i.e. they are in an epitaxial relationship to the dendritic arm. Analyzing the crystal orientation showed that one (100)-plane in the Y-ZrO_2_ is always parallel to the (001)-plane of the ε-YS dendrite.

**Figure 10:**
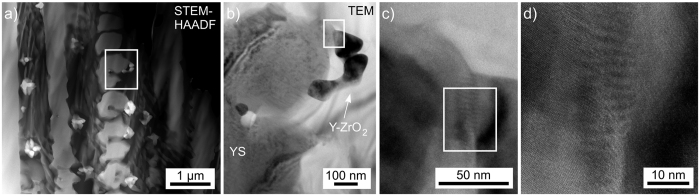
(**a**) STEM-HAADF micrograph of the same area shown in Fig. 5, the framed area is shown in greater detail in the TEM micrograph (**b**). The framed boundary between YS and Y-ZrO_2_ is again featured in greater detail (**c**) where the framed area is again presented in further detail (**d**).

The immediate contact area between one of the YS dendrites in Fig. 5 and an Y-ZrO_2_ crystal is presented in Fig. 10. The detailed Figures (c) and (d) clearly illustrate the direct contact between the crystals, however they are not aligned in a suitable orientation to perfectly describe the epitaxial relationship proposed in Fig. 9. Hence a cut plane transecting crystallization layer 1 *parallel* to the surface was prepared in order to test this orientation relationship in a larger area.

**Figure 11:**
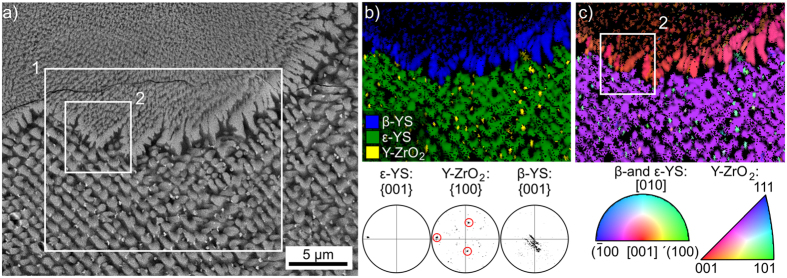
Cut plane parallel to the surface: (**a**) SEM-micrograph, (**b**) phase + IQ map and (**c**) IPF + IQ-map of an EBSD-scan performed in frame 1. Selected PFs of the respective phases within the scanned area are presented below. The area inside frame 2 is presented in greater detail in Fig. 12.

Figure 11 presents an SEM-micrograph featuring an ε-YS dendrite (bottom) with multiple Y-ZrO_2_ particles (bright) in the interdendritic spaces. It also highlights the boundary between the ε-YS dendrite and the β-YS area (top) of layer 2. Interestingly, Y-ZrO_2_ particles are not discernible in the β-YS area where the intercrystalline spaces are much smaller. An EBSD-scan was performed on the area in frame 1 with a step size of 125 nm and the phase + IQ-map of this scan is presented in Fig. 11(b). The IPF + IQ-map of the same scan is presented in Fig. 11(c) and selected PFs of the respective phases in the scan are presented. The ε-YS dendrite shows a single color in the IPF + IQ-map and the {001}-PF confirms the single orientation of this phase with the c-axis almost parallel to the surface of this cut plane. The data points attributed to Y-ZrO_2_ predominantly show a turquoise color in the IPF + IQ-map and the corresponding {100}-PF shows three dominant pole positions (circled in red) which belong to a single cubic orientation where a <100> -direction is parallel to the [001]-direction of the ε-YS dendrite. 63% of the Y-ZrO_2_ data points in this scan show this orientation, confirming the epitaxial relationship between ε-YS and Y-ZrO_2_ proposed in Fig. 9. The remaining Y-ZrO_2_ orientations show some variation of the dominant orientation via rotations of 45° around one of the <100> directions. This epitaxial relationship proves that these cubic crystals formed after the ε-YS dendrite and supports the conclusion in ref. 25 that Y-ZrO_2_ does not act as a nucleation agent in this glass-ceramic.

The {001}-PF of β-YS in Fig. 11 clearly shows an orientation preference, but it is not nearly as discreet as the previously described epitaxial relationship. At a first glance, the c-axes of the β-YS crystals are oriented perpendicular to the surface and hence parallel to the main growth direction of this phase in agreement with Fig. 5. However, the rotation of the c-axis around this primary texture is not symmetric and it seems that the c-axes show a greater degree of variation parallel to the ε-YS/β-YS boundary than perpendicular to it.

**Figure 12:**
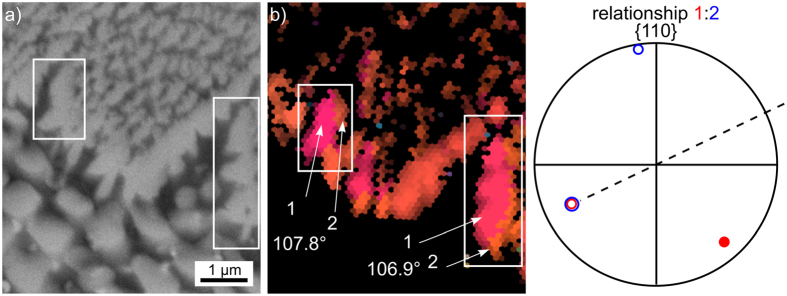
(**a**) SEM-micrograph and (**b**) IPF- + IQ-map containing only data points of β-YS of the area in frame 2 in Fig. 11. The relationship between the orientation domains 1 and 2 is illustrated in the {110}-PF and the misorientation angles between the respective domains are stated below.

Figure 12 presents the area in frame 2 in greater detail to show that the crystal structures at the phase boundary frequently do not show a grain boundary within one structure in the SEM-micrograph but multiple orientations. The misorientation angles and 110-poles of the orientation domains 1 and 2 in the framed areas (the c-axes of both domains are parallel) are presented to illustrate that the β-YS shows the same twinning relationship already observed in a different glass-ceramic where growth barriers forced the β-YS crystals into growth structures showing a five-fold pseudo symmetry^28^. The twinning relationship is characterized by a ≈108° rotation around the [001]-direction so that the twins share one parallel (110)-plane as confirmed in Fig. 12. More than 50% of the grain boundaries between β-YS domains in the featured EBSD-scan show a misorientation angle of 107 ± 5°.

**Figure 13:**
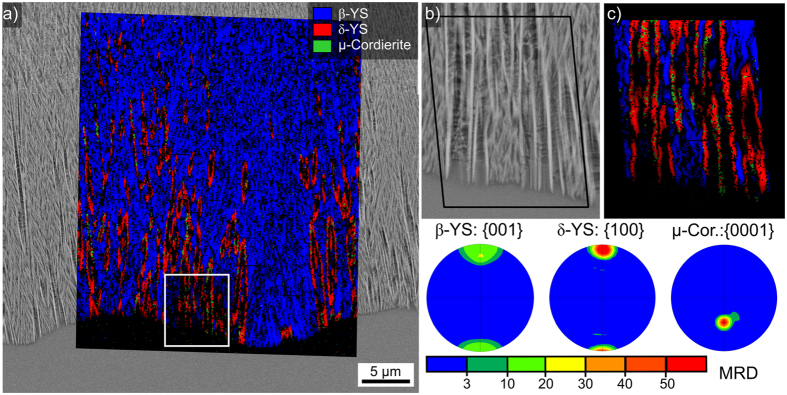
(**a**) SEM-micrograph of the growth front superimposed by the phase + IQ-map of an EBSD-scan performed on the area. The area framed in white is presented in greater detail in the SEM-micrograph (**b**). (**c**) Phase + IQ-map of the area framed in black. PFs of textures calculated from the data sets of β- and δ-YS as well as μ-cordierite in the large scan area are also presented.

The Figs 8, 9 and 11 show that β-YS forms a relatively compact layer adjacent to the ε-YS dendrites before fragmenting into finer growth structures where EBSD-pattern acquisition becomes problematic. This crystallization layer 2, where basically only β-YS and residual glass are detected, reaches a thickness of 570 μm after the two-step heat treatment of the samples featured in the Figs 8, 9 and 11. Figure 13 presents an SEM-micrograph of the growth front, below which uncrystallized glass remains in this incompletely crystallized glass-ceramic. The phase + IQ-map of an EBSD-scan performed on this area is superimposed on the micrograph to show that δ-YS is increasingly detected at this growth front, i.e. it probably formed at lower temperatures when the sample was cooled to RT. There is significantly more δ-YS at this growth front than at the growth fronts of samples only annealed at 950 °C. A few data points are again attributed to μ-cordierite, but as before single patterns reliably confirming the occurrence of this phase could not be acquired. The utmost growth front at the area framed in white is presented in greater detail in Fig. 13(b) and the phase + IQ-map of a more detailed EBSD-scan performed on the area framed in black is presented in Fig. 13(c). While the very fine grained structures are attributed to β-YS, the slightly brighter, straight structures are attributed to δ-YS. Considering the six-sided channels described in Fig. 6(b), it is plausible to assume that the close to parallel δ-YS structures in Fig. 13 are cross sections through such tubes. The presented PFs of textures calculated for β- and δ-YS from the large scan show the same, but more clearly developed, orientation preferences described for these phases in layer 1 (see Fig. 6): the c-axes of β-YS and the a-axes of δ-YS are preferably oriented parallel to the main growth direction and hence perpendicular to the initial sample surface. The texture of μ-cordierite, based on 1659 data points, is also presented and shows a high degree of orientation with the c-axes not truly parallel to the YS-domains. However, it is almost impossible that such a texture is the result of indexing errors. Although the latter also do not show a random orientation distribution, an intensity of 50 MRD would be very unusual. It should be noted that the existence of μ-cordierite at the growth front is quite possible because here the residual glass between the YS-structures is exposed to temperatures below the formation temperature of indialite when the samples are cooled. Hence it is quite likely that the μ-cordierite data points at the growth front are correctly indexed despite the lack of a single EBSD-pattern reliably indexable as μ-cordierite from these samples.

**Figure 14:**
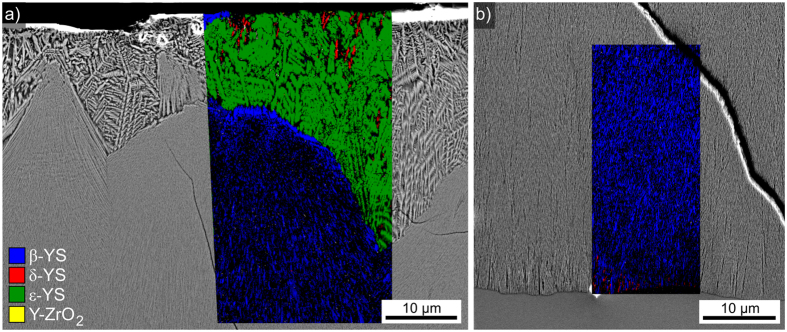
SEM-micrographs superimposed by phase + IQ-maps obtained (**a**) at the edge to the initial surface and (**b**) at the growth front of the cross section prepared from a sample directly heated to 1060 °C for 1 h.

If the glass is heated directly to 1060 °C and held for 1 h, the double layered crystallization described above is also observed as illustrated in Fig. 14.

Figure 14(a) features the crystallization adjacent to the initial sample surface while Fig. 14(b) presents the growth front after cooling. While dendritic ε-YS dominates layer 1 and β-YS dominates layer 2, the secondary phases, e.g. δ-YS, seem to occur less frequently in layer 1 as well as at the growth front. This observation is quite similar to that described for samples solely annealed at 950 °C in Fig. 7, so either the increased δ-YS content at the growth front in Fig. 13(a) is somehow caused by the two step annealing process or the cooling rate at the growth front was not truly identical. A slower cooling rate could e.g. allow the formation of more δ-YS and μ-cordierite.

**Figure 15:**
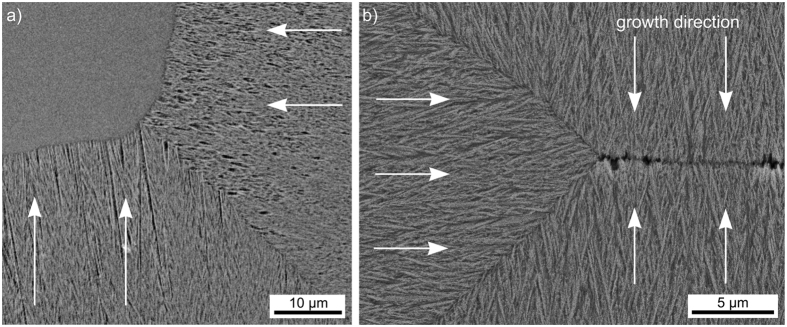
SEM-micrographs of growth front collision zones in (**a**) the partially crystallized sample also featured in Fig. 13, and (**b**) a fully crystallized sample where no uncrystallized glass remains in the bulk. The main growth direction in the respective growth domains is indicated by arrows.

Highly oriented crystal layers grown from the surfaces have been described in various glass compositions including the fresnoite system. Three principal interactions between such growing layers have been outlined^36^. While crystal growth is accelerated at the interaction zone of two colliding growth fronts during the growth of Sr-fresnoite^36^,^37^, no clear interaction was observed during the growth of Ge-fresnoite^38^. As illustrated above, crystallization layer 2 in the current system is mainly formed by the oriented growth of β-YS. Figure 15(a) presents an SEM-micrograph of the growth front interaction obtained from the same cut plane featured in Fig. 13, while the contact zone of growth fronts originating from the top, bottom and side of a completely crystallized sample is featured in Fig. 15(b). The main growth direction in the respective growth domains are illustrated by arrows. The rounded interface at the growth front interaction area in Fig. 15(a) shows that the crystal growth velocity is slightly larger. However, a spike between the top and bottom growth fronts comparable to that observed in the Sr-fresnoite^36^,^37^ system is not observed. EBSD-scans performed on these areas did not indicate any changes in the growth direction comparable to those observed in Sr-fresnoite^36^,^37^. However, while some δ-YS was detected at the growth fronts in Fig. 15(a), none was observed in the area featured in Fig. 15(b). This supports the conclusion that the δ-YS at the growth front in Fig. 13 grew during cooling, as also indicated by its different growth morphology, and is not the result of a phase transformation from β-YS to δ-YS.

**Figure 16:**
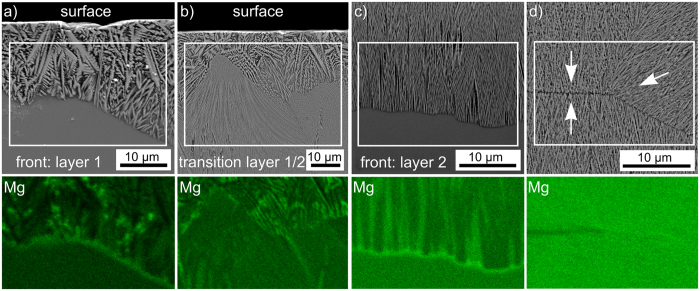
SEM-micrographs of (**a**) the growth front of layer 1, (**b**) the transition from layer 1 to layer 2, (**c**) the growth front of layer 2 and (**d**) the collision zone of growth fronts of layer 2. The arrows highlight the respective growth directions. EDS-maps of the relative Mg distribution in the framed areas are presented below the respective figures.

SEM-EDS-maps using an acceleration voltage of 10 kV were performed on the various growth fronts observed in these glass-ceramics. While most of the elements showed distributions in agreement with the detailed analysis presented in Fig. 5, Mg accumulations were detected at the immediate growth fronts of the crystallized layers 1 and 2. Figure 16 presents the results obtained from (a) the growth front of layer 1 including both zones 1 and 2 in the glass-ceramic also featured in Fig. 6. Figure 16(b) shows the transition from layer 1 to layer 2 which is representative for all glass-ceramics containing both layers, i.e. 10 h at 950 °C and any glass-ceramic heated to 1060 °C. Figure 16(c) features the growth front of layer 2 in a sample annealed via the two step crystallization also analyzed in the Figs 8, 9, 10, 11, 12, 13 while the growth fronts in Fig. 16(d) collided at 1060 °C and formed a comparably stable environment at this high temperature before cooling. An accumulation of Mg is clearly indicated at the growth fronts of both crystallization layers 1 and 2. In contrast, only a slight enrichment is observed at the transition from layer 1 to layer 2 and almost none where the growth fronts collided at high temperatures. This indicates that the Mg enrichment predominantly occurs when the sample is cooled at the end of the process if uncrystallized glass remains between the growth fronts. However, the slight enrichment at the transition from layer 1 to layer 2 indicates that ε-YS probably accumulates some Mg at the growth front. This probably triggers the formation of layer 2 by modifying either the local chemical composition and/or the local viscosity at the growth front to a preferred crystallization of β-YS which grows via a different mechanism and accumulates less Mg at the growth front.

## Discussion

The results presented above touch multiple aspects of glass crystallization:

### Growth Mechanisms

The different morphologies of the YS-phases at the immediate surface indicate that they grew independently and that phase transitions between the yttrium silicates did not occur. Additionally, the growth mechanism change of α-YS, i.e. from polygon (rhomboids) to dendritic growth with increasing annealing temperatures, is in agreement with established growth models linking these mechanisms to the growth velocity. For example, crystallites showing both polygon and dendritic growth at opposite ends have been observed in a glass-ceramic annealed at only one temperature due to an inhomogeneous chemical composition of the matrix^26^. A link of all known growth mechanisms via a specific growth velocity has been proposed in ref. 39, but a more detailed discussion of this topic is beyond the scope of this article.

### Cordierite Surface Crystallization

The described surface crystallization of cordierite is quite interesting because the surface crystallization of indialite in a glass with the stoichiometric indialite composition shows neither a texture nor any significant orientation change within the individual crystals^40^. By contrast, a systematic orientation change has been observed in indialite crystals grown in a boron doped MgO/Al_2_O_3_/SiO_2_ (MAS) glass^41^. Comparably small orientation changes and a topography were observed in indialite containing domains after crystallization in a related MAS system free of Y_2_O_3_^1^. It seems to be typical for indialite to vary its crystal orientation when faced with growth obstacles, i.e. diffusion barriers.

Taking into account that indialite changes its orientation during growth so that the c-axis becomes parallel to the main local growth direction, it seems logical that orientations with the c-axis parallel to the surface occur more frequently as the crystal lattice spreads along the surface. As it is unknown whether these indialite domains nucleated at or below the immediate sample surface, there is no way to be certain whether the observed orientation preference is caused by oriented nucleation, or rather if the orientation changes during growth, based on these measurements alone. However, considering that neither μ-cordierite (the low temperature phase of indialite) nor indialite were detected in the glass-ceramic annealed at only 900 °C (see pattern b) in Fig. 1 and the results presented in Fig. 2, the strong tendency of these cordierite phases to nucleate at the surface^1^,^40^,^41^ and the composition of the initial glass, the most logical explanation for these observations is that indialite was the last phase to crystallize. It probably grew along connected channels of residual glass formed by the growth of the YS-phases and thus filled large surface areas which do not appear to be connected at the immediate surface.

### Y_2_Si_2_O_7_-crystallization

Multiple yttrium silicates are simultaneously detected within one material in agreement with recent publications^25^,^26^,^27^,^28^,^29^, although the glass-ceramics presented here were never heated above the 1225 °C below which α-YS is stated to be the only stable phase^31^. Interestingly, δ-YS seems to preferably crystallize at lower temperatures than β-YS as it is predominantly formed during the early stages of crystallization or at growth fronts advancing while the glass-ceramics are cooled. β-YS preferably grows in the direction of its shortest crystallographic axis while δ -YS preferably grows in the direction of its longest crystallographic axis in this glass which is in agreement with the growth observed in the bulk of a YAG-containing glass ceramic^28^. In agreement with recent results, twinning is confirmed in α^29^- and β-YS^28^.

### Growth Model for this Glass-Ceramic

The crystallization of this glass begins with the almost simultaneous but independent formation of α-, β-, δ- and ε-YS at the surface while bulk nucleation is never observed. Although α-YS shows the largest number of crystallites when annealing at 900 °C, it is solely detected at the immediate surface of all glass-ceramics. By contrast, it was also detected in the bulk after crystallizing the parent glass^25^. When annealing at 950 °C or higher, dendritic ε-YS dominates the first layer of crystallization which shows two zones of crystal growth: zone 1 shows a complex microstructure containing relatively large quantities of β- and δ-YS in addition to ε-YS while zone 2 is almost solely occupied by ε-YS dendrites. As layer 1 grows, Mg is expelled into the interdendritic spaces of ε-YS, but it also accumulates at the growth front where the modified composition most probably triggers a secondary nucleation of β-YS with a {001}-plane preferably oriented somewhat perpendicular to the sides of a neighboring ε-YS dendrite. β-YS forms a thin layer of compact crystallization, only a few μm thick, before it separates into smaller crystal structures which then propagate through the remaining uncrystallized glass until either colliding with another growth front or the sample is cooled. A strong texture with the c-axes aligned to the primary growth direction is formed during crystallization. If the growth front is cooled before the sample is fully crystallized, varying amounts of δ-YS and probably μ-cordierite may be detected along with a clearly pronounced enrichment of Mg at the growth front.

While some ε-YS dendrites manage to penetrate more than 150 μm into the bulk, the large majority grow less than 50 μm before a thin layer of compact β-YS is observed. It seems plausible that some specific orientations of ε-YS allow a formation of dendrites with their primary and secondary structures aligned to the Mg-enriched growth front in a specific orientation so that part of the Mg-enriched glass is regularly incorporated into the interdendritic spaces. This would delay the accumulation of Mg and hence allow some ε-YS dendrites to grow further into the bulk than most others.

Additionally, the β-YS containing crystallization zone 1 in layer 1 generally extends further into the bulk than zone 2, indicating that β-YS grows faster than the dendritic ε-YS. However, β-YS can obviously only grow a few μm as a compact layer before fragmenting into the fine microstructure of layer 2, which shows some similarities to the seaweed structure described to occur when dendritic growth is increasingly undercooled^42^. Perhaps the ability of β-YS to change its growth direction via the low-energy mechanism of twinning enables a continuous circumvention of small amounts of the Mg-enriched glass at the growth front in contrast to the dendritic structures of ε-YS. A similar effect has been proposed to cause the texture described in Sr-fresnoite where residual SiO_2_ is finely dispersed between the textured crystals^36^.

As the YS-phases grow, the residual glass is enriched in Al, Mg and Zr which leads to the crystallization of the secondary phases Y-ZrO_2_, indialite and probably μ-cordierite. This is most impressively observed inside the ε-YS dendrites which show the largest intercrystalline spaces. Here, enough ZrO_2_ is accumulated to allow the formation of Y-ZrO_2_ which shows the dominant epitaxial relationship with the ε-YS-dendrite described in Fig. 11 in the bulk. The remaining residual glass approaches the chemical composition of cordierite, which enables the crystallization of indialite and probably μ-cordierite at the growth front during cooling. The nucleation rate of indialite is very low (probably only 17 nuclei managed to grow in the area of Fig. 3) but once an indialite domain grows, it may spread along the channels of residual glass, optimizing its orientation, to form circular, crystallographically linked areas of several hundred μm diameter (Fig. 3). It seems likely that the domains of residual glass in the microstructure of layer 2 are simply too small to allow the formation of any crystals or at least their detection in the SEM. Hence this glass basically shows the reversed order of crystallization observed in the parent glass^25^ although spinel was not detected in the glass-ceramics analyzed here (which, however, may be due to a peak superposition in the XRD-patterns of Fig. 1).

Concerning the crystal orientations observed at the immediate surface, the textures presented in Fig. 4 indicate an oriented nucleation of ε-YS with the c-axis preferably parallel to the surface and of Y-ZrO_2_ with a {111}-plane parallel to the surface. Oriented nucleation has currently been detected in more than five glass systems, most recently in Ge-fresnoite^43^ where multiple phases showed up to three orientation preferences at the immediate surface after crystallization^43^. As the ε-YS dendrites in the EBSD-scan of Fig. 4(a) are not aligned, but the {111}-PF shows a basically random rotation around the central 111-pole, the Y-ZrO_2_ at the immediate surface nucleated independently from the ε-YS dendrites. Hence it seems logical to assume that Y-ZrO_2_ shows multiple relationships with the various interfaces in this glass-ceramic, even though only the one with ε-YS could be described in detail due to the occurring crystal sizes. The orientation preference of ε-YS does not confirm the texture indicated by the exaggeration of the 0nn-peaks in ref. 25, so the latter may be attributed to the peak superposition of multiple phases instead of a crystallographic texture. δ-YS grows in the form of needles at the surface and channels (i.e. hollow needles) in the bulk. It is predominantly observed at the immediate surface, in the zone 2 of layer 1 and at the growth fronts of incompletely crystallized samples, i.e. it predominantly forms in a dynamic environment such as heating, cooling, or where β- and ε-YS compete for the available space. As all δ-YS structures measured at the immediate surface show orientations with a [100]-direction parallel to the surface, an oriented nucleation of this phase is also very likely.

During growth into the bulk, all phases develop an orientational preference with their fastest growing direction parallel to the primary direction of growth. This leads to a high orientation of β-YS in layer 2 which could be grown to very large thicknesses due to the lack of bulk nucleation.

In summary, the crystallization behavior of the glass with the mol% composition 54.7 SiO_2_·10.9 Al_2_O_3_·15.0 MgO·3.4 ZrO_2_·16.0 Y_2_O_3_ basically shows the inverted order of crystallization observed in the parent glass with the mol% composition 50.6 SiO_2_·20.7 MgO·20.7 Al_2_O_3_·5.6 ZrO_2_·2.4 Y_2_O_3_, where it was detected as the residual glass after crystallization. The almost simultaneous but independent nucleation of α-, β-, δ-, and ε-YS at the surface is followed by growth into the bulk, where ε-YS quickly dominates a first layer of crystal growth. An accumulation of Mg at the growth front probably triggers a secondary nucleation of β-YS, which forms a thin compact layer before fragmenting into a highly oriented layer of fine grained crystals occupying the remaining bulk. The residual glass between the YS growth structures allows the crystallization of indialite, yttrium stabilized ZrO_2_ (Y-ZrO_2_) and very probably μ-cordierite during cooling. An epitaxial relationship between Y-ZrO_2_ and ε-YS is proven and multiple twinning relationships occur in the YS phases.

## Methods

A glass of the composition 54.7 SiO_2_·10.9 Al_2_O_3_·15.0 MgO·3.4 ZrO_2_·16.0 Y_2_O_3_ was prepared from the raw materials SiO_2_, 4 MgCO_3_·Mg(OH)_2_·5 H_2_O, Al(OH)_3_, and Y_2_O_3_. Quantities of 200 g of this glass were melted in a platinum crucible at a temperature of 1590 °C and held for 2 h. The melt was cast into water, subsequently dried, and finally crushed into pieces with sizes ≤1.25 mm. The glass was re-melted in order to improve the homogeneity at 1590 °C for another 2 h and then cast into a steal mould preheated to 600 °C. It was then transferred to a cooling furnace preheated to 850 °C which was subsequently switched off to allow a slow cooling of the glass to room temperature with a rate of approximately 2 K min^−1^. The glass was cut into pieces, polished with decreasing grain sizes down to 0.25 μm and subsequently crystallized at temperatures from 900–1060 °C with a heating rate of 5 K min^−1^.

X-ray diffraction (XRD) was performed using Cu *K*_*α*_–radiation in a SIEMENS *D5000* diffractometer in a θ–2θ arrangement from 2θ = 10–60° with a step width of 0.2°.

In order to perform SEM studies, the samples were contacted with Ag-paste and coated with a thin layer of carbon at about 10^−3^ Pa to avoid surface charging. Some glass-ceramics were embedded in a polymer and cut in specific planes which were then polished with abrasive slurries down to diamond paste of 0.25 μm grain size before applying a final finish of 30 min using colloidal silica.

SEM analyses were performed using a scanning electron microscope (SEM Jeol*JSM 7001F*) equipped with an EDAX Trident analyzing system containing a Digiview 3 EBSD-camera. EBSD-scans were performed using a voltage of 20 kV and a current of ca. 2.40 nA. The scans were captured and evaluated using the software TSL OIM Data Collection 5.31 and TSL OIM Analysis 6.2. Unreliable data points were removed in all datasets used for orientation analyses by applying a Confidence Index (CI) filter of 0.1 after performing a grain CI standardization except for the data concerning ε-YS which showed a pseudo-symmetry problem. No further cleanups which actually modify orientations were applied. Pole figures of textures are presented in multiples of a random distribution (MRD).

High resolution (HR)-TEM and STEM analyses of the glass-ceramics were performed with an FEI Titan^3^ 80–300 electron microscope using an acceleration voltage of 300 kV. STEM images were obtained using a high-angle annular dark field detector (HAADF, Fischione Model 3000). EDXS was performed using a Super-X EDX detector equipped with four SDD detectors (FEI company) to obtain element distribution mappings with the software *Esprit* (Bruker company). Element mappings were derived by evaluating the lateral distribution of the peak intensity, i.e. the area underlying the *K*_*α*_ edges of the analyzed elements, with an automatic routine provided by the software. The STEM sample preparation was done by a purely mechanical wedge-polishing routine (polishing system Multiprep^TM^, Allied company), followed by a low-energy (2.5 keV) Ar^+^ broad beam final milling step (precision ion polishing system PIPS, Gatan company) to achieve electron transparency as well as to remove any residues from the mechanical polishing.

## Additional Information

**How to cite this article**: Wisniewski, W. *et al*. Surface Crystallization of a MgO/Y_2_O_3_/SiO_2_/Al_2_O_3_/ZrO_2_ Glass: Growth of an Oriented β-Y_2_Si_2_O_7_ Layer and Epitaxial ZrO_2_. *Sci. Rep.* **7**, 44144; doi: 10.1038/srep44144 (2017).

**Publisher's note:** Springer Nature remains neutral with regard to jurisdictional claims in published maps and institutional affiliations.

## Figures and Tables

**Table 1:** Notations of YS-phases and database entries correlated with them due to matching crystallographic data.

Y_2_Si_2_O_7_ type+database files	symmetry	space group (number)	lattice parameters [Å]			angles [°]			Ref.
			a	b	c	α	β	γ	
**α-type**	triclinic	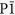 (2)	6.584	6.643	12.390	93.65	89.81	91.17	30
JCPDS 38-0223	triclinic	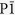 (2)	6.590	6.640	12.250	94.00	89.20		
ICSD 164148	triclinic	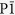 (2)	6.588	6.639	12.031	94.48	90.95	91.80	
ICSD 173383	triclinic	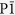 (2)	6.586	6.628	12.027	94.47	89.07	88.13	
**β–type**	monoclinic	C2/m (12)	6.845	9.139	4.687		100.57		30
JCPDS 38-0440	monoclinic	C2/m (12)	6.875	8.970	4.721		101.74		
ICSD 281312	monoclinic	C2/m (12)	6.869	8.960	4.716		101.73		
ICSD 281313	monoclinic	C2/m (12)	6.867	8.959	4.717		101.72		
**γ–type**	monoclinic	P2_1_/a (14)	5.610	10.833	4.690		95.72		30
JCPDS 42-0167	monoclinic	P2_1_/a (14)	5.579	10.857	4.696		95,99		
ICSD 28212	monoclinic	P2_1_/a (14)	5.540	10.780	4.66		96.10		
ICSD 164147	monoclinic	P 2_1_/c (14)	4.688	10.840	5.582		96.03		
**δ–type**	orthorh.	Pnam (62)	13.671	5.017	8.015				30
JCPDS 76-0204	orthorh.	Pnam (62)	13.665	5.016	8.139				
JCPDS 42-0168	orthorh.	Pnam (62)	8.152	13.660	5.020				
ICSD 33721	orthorh.	Pnam (62)	13.665	5.016	8.139				
**ε–type**	monoclinic	P2_1_/m (11)	7.503	8.063	5.022		112.05		30
JCPDS 74-1994	monoclinic	P2_1_/m (11)	7.500	8.060	5.020		112.00		
ICSD 28004	monoclinic	P 2_1_/m (11)	7.503	8.063	5.022		112.05		
**ζ–type**	monoclinic	P2_1_/m (11)	5.034	8.064	7.326		108.63		30
ICSD 416573	monoclinic	P2_1_/m (11)	5.036	8.064	7.326		108.63		
**η–type**	triclinic	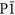 (2)	6.629	6.584	35.916	91.090	94.53	91.73	31
ICSD 173268	triclinic	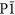 (2)	6.629	6.584	35.916	91.090	94.53	91.73	
**z-type**	n.A.	n.A.	n.A.	n.A.	n.A.	n.A.	n.A.	n.A.	32
JCPDS 21-1459	n.A.	n.A.	n.A.	n.A.	n.A.	n.A.	n.A.	n.A.	
